# 682. Novel *rpoB* mutations and trends in clinical *Clostridioides difficile* isolates with reduced fidaxomicin susceptibility

**DOI:** 10.1093/ofid/ofad500.744

**Published:** 2023-11-27

**Authors:** Anne J Gonzales-Luna, Thanh Le, Taryn A Eubank, Md Ekramul Karim, M Jahangir Alam, Khurshida Begum, Kevin Garey

**Affiliations:** Department of Pharmacy Practice and Translational Research, University of Houston College of Pharmacy, Houston, Texas, USA, Houston, TX; Department of Pharmacy Practice and Translational Research, University of Houston College of Pharmacy, Houston, Texas, USA, Houston, TX; University of Houston, Houston, Texas; Department of Pharmacy Practice and Translational Research, University of Houston College of Pharmacy, Houston, Texas, USA, Houston, TX; Department of Pharmacy Practice and Translational Research, University of Houston College of Pharmacy, Houston, Texas, USA, Houston, TX; Department of Pharmacy Practice and Translational Research, University of Houston College of Pharmacy, Houston, Texas, USA, Houston, TX; Department of Pharmacy Practice and Translational Research, University of Houston College of Pharmacy, Houston, Texas, USA, Houston, TX

## Abstract

**Background:**

Fidaxomicin use is increasing to treat *Clostridioides difficile* infection (CDI) following clinical treatment guideline updates placing it as first-line therapy. This puts a selection pressure on the development of fidaxomicin resistance, which has been associated with *in vitro* fitness costs compared to wildtype isolates. Here we aimed to assess the epidemiology of reduced susceptibility (RS) to fidaxomicin and prevalence of *rpoB* mutations in a clinical cohort.

**Methods:**

Stool samples from patients with CDI were collected from two different healthcare systems in Houston, TX between 2016-21. Fidaxomicin minimum inhibitory concentrations (MIC) were determined via agar dilution susceptibility testing following CLSI standards. RS was defined as MIC > 0.5 mg/L based on the EUCAST [T]ECOFF. A subgroup of 40 isolates with RS underwent Sanger sequencing to detect *rpoB* single nucleotide polymorphisms (SNPs).

**Results:**

Of 535 patients included, 114 (21.3%) had infecting strains with fidaxomicin RS (MIC50 = 0.5 mg/L, MIC90 = 1.0 mg/L). Fidaxomicin RS was more common in RT 027 (49.4%, 41/83) compared to non-027 strains (16%, 68/425; P < 0.001). There were no differences in the proportions demonstrating RS across collection years (P=0.18) or based on location in or out of the Texas Medical Center (P=0.29). Mean MICs were similar between isolates collected before and after the IDSA/SHEA guideline update in 2018 (P =0.1). *rpoB* SNP mutations were detected in 12.5% (5/40) of isolates at 7 novel base positions; no SNPs were detected at previously reported position 3428.

**Conclusion:**

RS to fidaxomicin occurred in one fifth of clinical isolates with no changes in incidence between 2016-21. Several new *rpoB* SNPs were observed in isolates with RS. Studies are needed to determine potential association(s) between fidaxomicin exposure and MIC changes.

**Disclosures:**

**Anne J. Gonzales-Luna, PharmD, BCIDP**, Cidara Therapeutics: Grant/Research Support|Ferring Pharmaceuticals: Personal Fees|Paratek Pharmaceuticals: Grant/Research Support|Seres Therapeutics: Grant/Research Support **Kevin Garey, PharmD, MS, BCIDP**, Acurx Pharmaceuticals: Grant/Research Support|Cidara Therapeutics: Grant/Research Support|Ferring Pharmaceuticals: Advisor/Consultant|Paratek Pharmaceuticals: Grant/Research Support|Seres Therapeutics: Grant/Research Support|Vedanta Therapeutics: Grant/Research Support

